# 'Wagon-wheel' mask as a tool to study anisotropy of porous silicon formation rate

**DOI:** 10.1186/1556-276X-7-421

**Published:** 2012-07-27

**Authors:** Ekaterina V Astrova, Yuliya A Zharova

**Affiliations:** 1 Department of Solid State Electronics, Ioffe Physical Technical Institute Russian Academy of Sciences, Politekhnicheskaya 26, St. Petersburg, 194021, Russia

**Keywords:** Mesoporous Si, Anodization, Wagon-wheel mask, Anisotropy

## Abstract

Relationship between the rate of electrochemical formation of mesoporous Si and the crystallographic directions has been studied by local anodization of wafers through a mask having the form of narrow long wedges radiating from the center in all directions (‘wagon-wheel’ mask). The etching rates were found from the side etching under the thin transparent n-Si mask. On p^+^-substrates of various orientation diagrams characterizing the distribution of pore formation rates over different directions in the wafer plane were constructed for the first time. The wagon-wheel method was applied to study the current dependence of the anisotropy. It was found that the orientation-related difference between the pore formation rates is 5% to 25%, depending on the crystallographic direction and the etching current density. At a current density of approximately 9 mA/cm^2^, the etching rates are related as *V*100:*V*113:*V*110:*V*112:*V*111 = 1.000:0.900:0.836:0.824:0.750. A general tendency is observed toward weakening of the anisotropy with increasing current. The highest rate always corresponds to the <100 > direction, and the rate ratio between the other directions varies with increasing current, which leads to a change of their sequence.

## Background

Analysis of the pore formation rate in relation to a crystallographic direction is important both from the standpoint of fundamental science, for understanding the nature of the porous structure formation, and from the practical standpoint, for solving various technological tasks based on utilization of porous silicon (por-Si) [1-5] or for creating a birefringent optical medium based on nanostructured silicon [6,7]. It is known that individual pores in meso-porous silicon form a dense array with a stable front propagating in the bulk of the crystal. Despite that individual pore channels in this array display strong anisotropy of their motion (microscopic anisotropy), the anisotropy in the propagation of the pore front (macroscopic anisotropy) is rather weak. Porous silicon has been intensively studied in the last two decades; however, the information about the macroscopic anisotropy of the electrochemical etching rate is scarce [8-12], and its quantitative characteristics are at all lacking. A possible reason is that no appropriate method of study has been developed so far. Recently, we suggested the method of local anodization of p-Si wafers through a mask in the form of narrow long wedges radiating from the center in all directions (‘wagon-wheel’ mask) [13], previously used to examine only the chemical etching anisotropy for Si in alkaline solutions [14,15]. The under-etching for this mask depends on the motion rate of the porous-layer boundary along different crystallographic directions lying in the sample plane and makes it possible to determine quantitative parameters of the anisotropy.

The present study is concerned with the anisotropy of pore formation in heavily doped (degenerate) p^+^-Si. The wagon-wheel procedure is used to construct diagrams of the etching rate distribution over different crystallographic directions, and the orientation dependence of the motion rate of the pore front is analyzed in relation to the etching current.

## Methods

**Figure 1:**
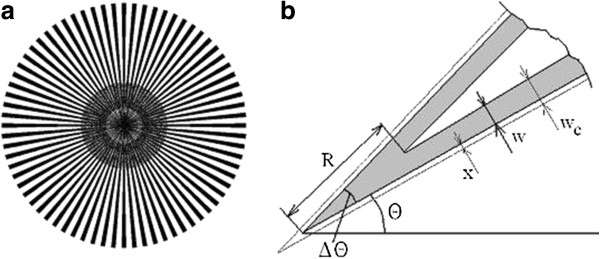
**Wagon-wheel mask.** (**a**) General view and (**b**) scheme illustrating the relationship between the mask under-etching *w* and the distance *R* from the mask center to the meeting point of pore fronts.

Let us consider the principle of the method [15]. The general view of the mask is presented in Figure 1a. The regions protected from the etching are isosceles triangles with a small angle *Δθ* at the vertex. These triangles radiate as beams from the center in different directions *θ* with the same angular step. Figure 1b shows one of these triangles. Usually, the mask under-etching is the same on both sides and equals *w*(*θ*)*.* The meeting position of the under-etch fronts can be found as *w = R* sin(*Δθ/*2). Using a mask with small angles *Δθ*, we can obtain *R* many times exceeding *w*. Because the anisotropy in porous layer formation is not strong [13], this increase in sensitivity is particularly important for reliably recording the effect. The dependence *R*(*θ*) reflects the angular distribution of the under-etching rates: the larger *R*(*θ*), the higher the rate of pore propagation in the perpendicular direction, i.e., *w*(*θ*) is proportional to *R* (*θ −* 90°).

**Figure 2:**
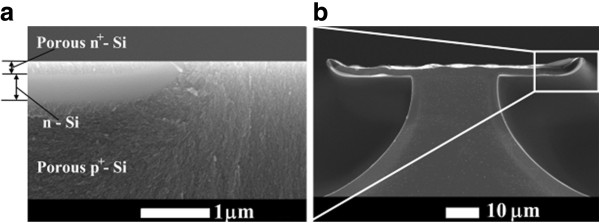
**Cross-sectional SEM images of a sample with the diffusion n-Si mask.** (**а**) Upon anodization and (**b**) upon dissolution of porous silicon. Rectangular area at the upper right side of (b) corresponds to the magnified image in (a).

**Figure 3:**
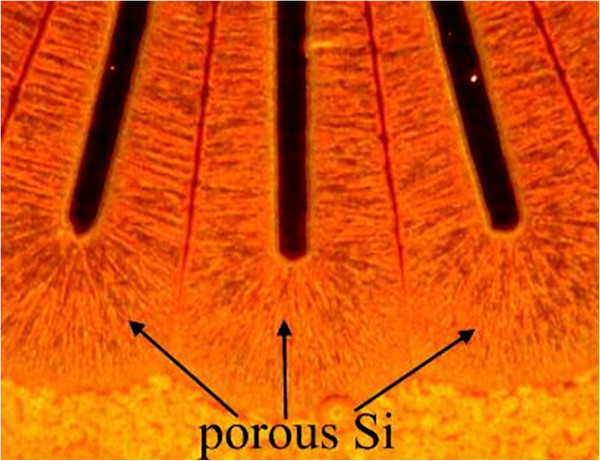
**Current-oriented pores under the mask in a p**^**−**^**sample: plan view, optical microscope, dark field.**

**Figure 4:**
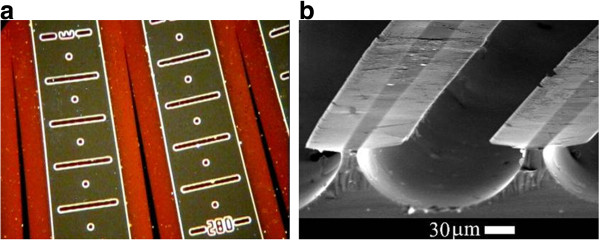
**Fragment of a sample anodized via the wagon-wheel mask.** (**a**) Plane view optical image (dark field mode), under the mask por-Si is reddish; (**b**) cross-sectional SEM image upon dissolution of por-Si.

The structure of the meso- and micro-porous layers on a p-type substrate is characterized by current-oriented pores [16]. This means that the general direction of the pore ensemble propagation follows the current lines, and the electric field is the main driving force for the mask under-etch. The evidence of the current-oriented pores can be seen in the cross-sectional scanning electron microscopy (SEM) image (Figure 2a) and in the plan-view optical image in Figure 3. It should be noted that, when determining from *w* the pore formation rate in the tangential directions parallel to the substrate plane, we neglect the possible influence exerted on this rate by the front of the electrochemical process, which freely propagates deep into the crystal and is nonuniform in its characteristics (for more details, see [13]). In contrast to the alkaline chemical etching, the formation of porous silicon in HF electrolytes is characterized by a low anisotropy. That is why the faceting does not noticeably modify the shape of porous regions etched through a mask. The boundary between porous Si and the substrate is usually rather rounded, and the upper part of the etching front is vertical (normal to the wafer surface) (see Figure 4b). This means that the in-plane under-etching length *w* correctly characterizes the etching rate in the direction perpendicular to the mask beam.

In the first experiments with the wagon-wheel mask [13], the transparence of the mask was of minor importance because, to visualize *R*(*θ*), the sample was etched in KOH to dissolve por-Si and then the mask was mechanically removed over the merged pore fronts. In the present study, the porous layer was not dissolved. The distance *R*(*θ)* was found from the position of the wedge vertex well seen under the optical microscope (Figure 4a). For this to be done, we need a thin transparent mask. In addition, application of the wagon-wheel technique to electrochemical etching of Si requires a mask resistant to HF under anodization. Our choice is based on the dependence of the I-V curves for Si-electrolyte on the dopant type and concentration [17]. The sequence of the curves for each type of doping is shifted to the right in the general order:

(1) $$n^{+}<p^{+}<p<n\text{.}$$

We used this potential shift to selectively form pores in differently doped areas.

The starting material in our experiments was p^+^-Si. (111), (100), and (110) wafers were cut from the same ingot doped with boron to a concentration of 2 × 10^19^ cm^−3^ (6 mΩ⋅cm). To form the mask for local anodization, we formed n-Si layers either by local diffusion of phosphorus at 1,100 °C (p-n junction depth *x*_*j*_ = 3 μm) or by ion implantation (dose 5 × 10^15^ cm^−2^) with subsequent annealing (*x*_*j*_ ≈ 1.4 μm). The surface concentration of phosphorus was *N*_*s*_ = 10^20^ cm^−3^. The local doping with phosphorus was performed using thermally grown SiO_2_ film prepatterned by photolithography.

The wagon-wheel mask EA-3 had the form of 72 beams with an angular width *Δθ* = 2° radiating with a step *θ =* 5°. The beam length was 1 cm; the full anodization area, 3.14 cm^2^, of which the unmasked part constituted 60%, i.e., 1.88 cm^2^. The anodization was performed in a 1:1 HF-ethanol electrolyte in the galvanostatic mode at *I* = const for 7 to 120 min to a depth of approximately 50 μm. This corresponds to a change in the current density from *j*_0_ at the start (*t* = 0) to *j*_*f*_ = 0.65 *j*_0_ by the end of the process. The anodization rate was found as *V*(*θ*) *= w*(*θ*)/*t*, where *t* is the etching duration*.* Experiments on the current dependence were performed with (110) wafers. In these experiments, the product *I⋅t* was kept constant. To enable reading of *R*(*θ*), the mask was provided with a scale whose divisions indicated the distance *R* from the mask center and the angle *θ* (see Figure 4a). The scale marks were formed by n-Si strips and points on the beams opened for anodization. To find the under-etch length for the mask produced by diffusion, we introduced a correction for the increase in the n-type beam width *x* due to the side diffusion of phosphorus, *w*_*c*_ *= w + x* (see Figure 1b), where *x* = 0.8*x*_*j*_. In the case of ion implantation, this correction is negligible and can be disregarded.

## Results and discussion

It can be seen in Figure 2a that the upper part of the mask, heavily doped with phosphorous, becomes porous, with only the deeper part remaining intact. This agrees with inequality (1) when n^+^ is preferably etched. As soon as the etching front reaches n-Si, p^+^-Si substrate becomes predominantly etched. For this reason, the n-Si prevents anodization. The formation of the porous material from the upper layer of n^+^-Si results in upward-bent mask edges in Figure 2b, formed upon dissolution of porous silicon. The phosphorus concentration in the region of lateral diffusion beneath the oxide mask is lower than that in the opened window, and therefore, this part of n-Si at the mask edge was passivated under anodization. As for the layer ion-implanted with phosphorus, we found that it works upon a high temperature treatment at 1100 °C for 10 min, when the moderately doped n-Si becomes thick enough. Both masks are transparent to the visible light of the microscope. We found that the dark field mode is preferable.

**Figure 5:**
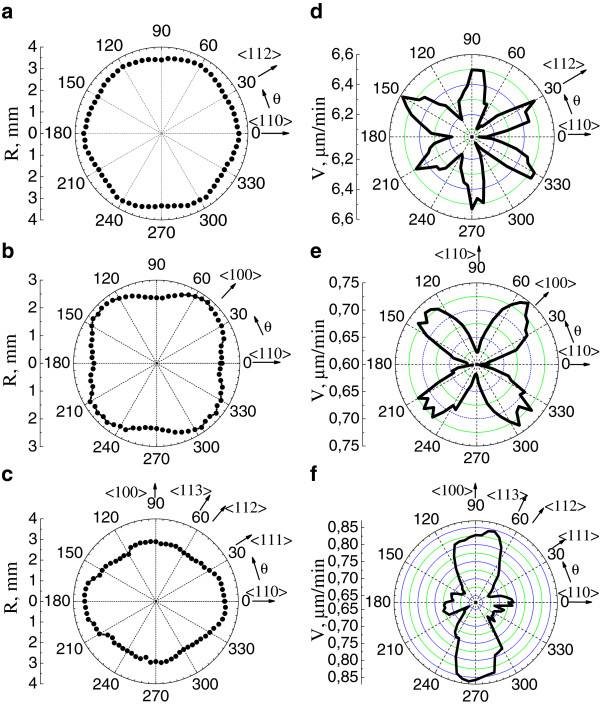
**Angular dependence in polar coordinates.** For *R*(*θ*) (**a,b,c**) and for the corresponding etching rates *V = f*(*θ*) (**d,e,f**), obtained on a (111) wafer (a and d) etched at a current *I* = 800 mA for *t* = 10 min and on (100) (b and e) and (110) (c and f) wafers etched at *I* = 50 mA for *t* = 70 min.

Figure 5a,b,c shows the dependences *R*(*θ*) plotted in polar coordinates for (111), (100), and (110) wafers. The patterns obtained are close to a hexagon, square, and ellipse, respectively. Figure 5d,e,f shows the corresponding etching rate diagrams *V*(*θ*). It should be noted that the *V*(*θ*) curve is turned by 90° relative to *R*(*θ*) in the same coordinate system based on the crystallographic directions. The plots are constructed so that the <110 > axis in all cases runs in the horizontal direction and corresponds to the angle *θ* = 0°. It follows from the plot in Figure 5d that the etching rate in the <112 > directions exceeds that for the <110 > directions (*V*112 > *V*110). The diagram in Figure 5e indicates that the rate of pore formation along <100 > is higher than that for <110 > (*V*100 > *V*110). The relative etching rates can be studied most conveniently for numerous directions using (110) wafers. The etching rates can be determined from Figure 5f simultaneously for five low-index axes <110>, <111>, <112>, <113 >, and <100>. It was found that the ratio between the etching rates strongly depends on the etching current density, which affects the shape of the diagram.

**Figure 6:**
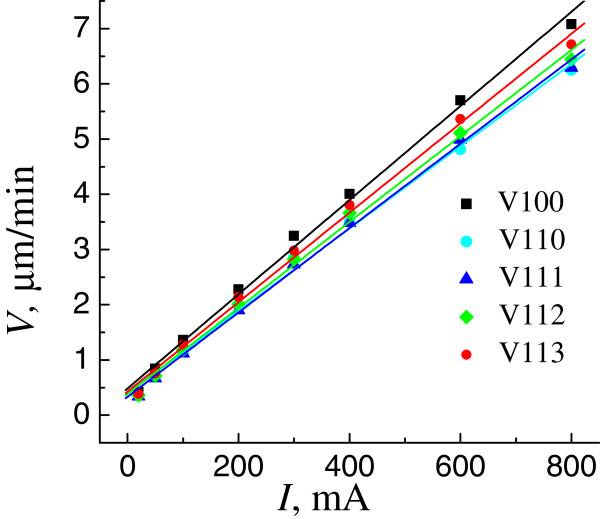
Motion rate of the pore front in different crystallographic directions vs. the etching current.

**Figure 7:**
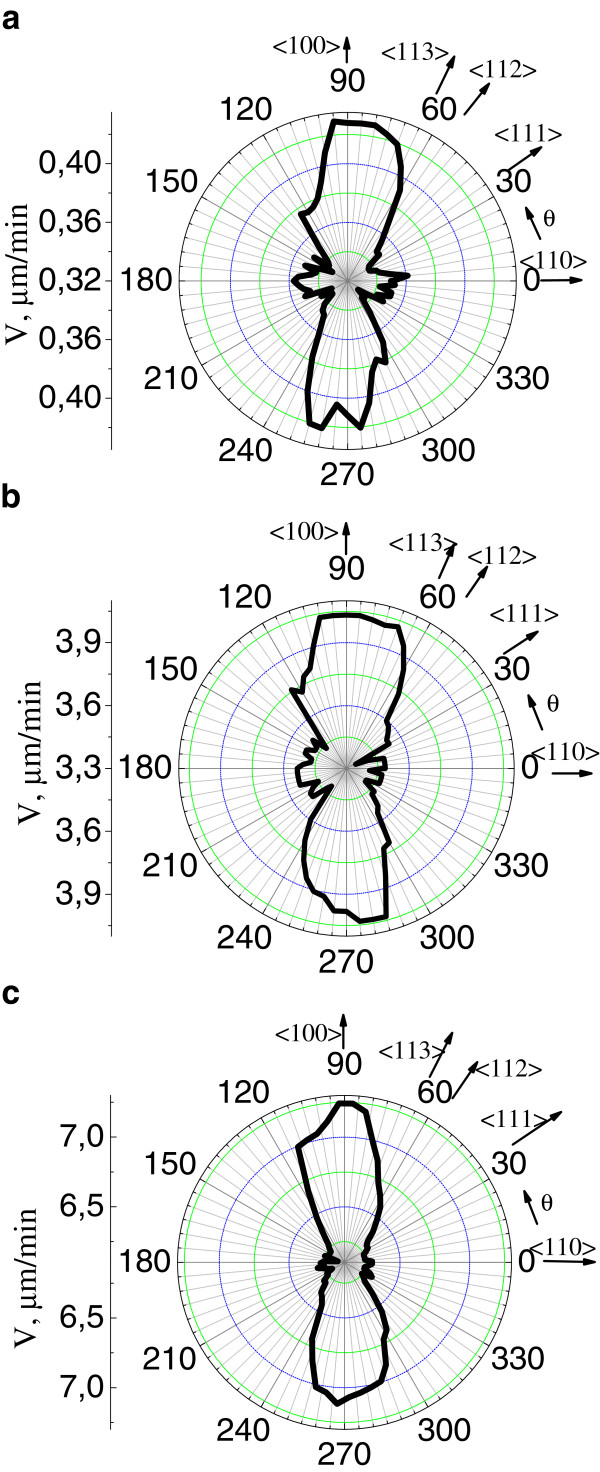
**Variation of the etching rate diagram for (110) wafers with increasing current. ***I* = (**a**) 20, (**b**) 400, and (**c**) 800 mA.

To find the etching rates at a prescribed current, we averaged values of *V* over two or four equivalent crystallographic directions. The current dependences of the etching rates in Figure 6 show that, for all the directions, the etching rate grows with increasing current. The experimental points are well approximated with a linear dependence having the largest slope for the <100 > direction. At all currents in the range *I* = 20 to 800 mA (*j*_0_ ≈ 8 to 400 mA/cm^2^), the highest etching rate corresponds to the <100 > direction. The same orientation is the most sensitive to changes in current. Figure 7 shows etching rate diagrams for a (110) wafer for three different currents. It can be seen that the pattern determining the rate distribution is mostly transformed due to a decrease in the rate *V*110 relative to the rates along other directions.

The anisotropy was evaluated by parameter *А*, the ratio of the etching rate in a certain direction to the maximum rate along <100>, *A*_110_ = *V*110/*V*100, *A*_111_ *= V*111/*V*100, etc. It is noteworthy that the larger *A*, the weaker the anisotropy. Table 1 lists numerical values of parameter *А* at currents corresponding to the rate diagrams for the (110) plane in Figures 5f and 7a,b,c. Also presented are approximate values of the average current density *j*_av_ in anodization in these modes.

**Table 1:** **Parameter *A *for different crystallographic orientations and etching currents**

***I *(mA)**	***j***^**a**^_**av **_**(mA/cm**^**2**^**)**	**<110>**	**<111>**	**<112>**	**<113>**	**<100>**
20	9	0.836	0.750	0.824	0.900	1
50	22	0.846	0.786	0.850	0.914	1
400	175	0.868	0.870	0.900	0.942	1
800	350	0.870	0.882	0.905	0.946	1

^a^*j*_av_ *=* (*j*_0_ *+ j*_*f*_)/2 = 0.825 *j*_0_.

**Figure 8:**
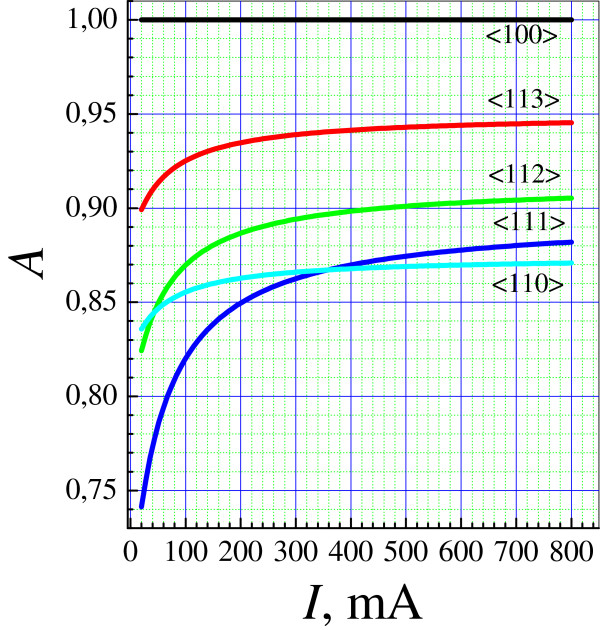
**Parameter *A *vs. the etching current.** Etching rates in different directions are normalized to the rate along the <100 > axis.

Figure 8 shows *А*(*I*) dependences obtained, as also those in the table, using the linear approximation of *V*(*I*) represented by solid lines in Figure 6. It can be seen that the values of *А* fall within the range 0.75 to 0.95, which indicates that the macroscopic anisotropy of mesoporous silicon formation rate may be as strong as 25%. The anisotropy related to the fastest crystallographic direction <100 > is the most pronounced at low currents and becomes weaker with increasing current.

Two intersection points of the curves *A*(*I*) can be seen in Figure 8. The intersection of *A*_111_ and *A*_110_ means that at currents *I* < 350 mA the minimum anodization rate is observed for the <111 > direction, and at *I* > 350 mA for the <110 > direction. Near the second intersection point of the curves for *A*_112_ and *A*_110_ at *I ≈* 40 mA, the motion rates of the pore front along the <112 > and <110 > directions become approximately the same, and anodization of Si (111) wafers in this mode yields a diagram on which preferred directions cannot be distinguished. Therefore, it was necessary to use a high etching current for recording an anisotropy on (111) wafers (see Figure 5a,d). The ratio between the rates *V*110 and *V*100 experiences the minimal variation over the whole range of currents under study. Therefore, the rate diagram for (100) wafers in the form of four petals could be obtained at all the currents.

**Figure 9:**
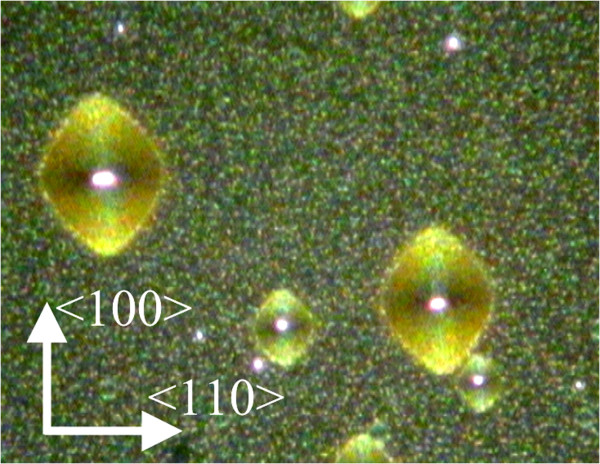
**Patterns formed on a (110) wafer.** The result of propagation of the pore front beneath the n-Si mask from small openings.

Previously, attempts have already been made to use local etching through a mask for studying the macroscopic anisotropy of pore formation [9,11,12]. This was done by fabricating a cross-sectional cleavage and analyzing the shape of the Si - por-Si front. However, this proved to be rather labor-consuming and furnished only qualitative data on the anisotropy. The suggested wagon-wheel method is based on an analysis of the mask under-etching, i.e., on an examination of the projection of the porous front on the wafer plane. This analysis became possible because of the transparence of the mask used in electrochemical etching. Generally speaking, the etching rate anisotropy can be recorded under a microscope upon anodization through a mask with the simplest configuration. As an example, we can mention oval patterns in Figure 9, elongated along the ‘fast’ <100 > direction and formed by the por-Si front propagating from small openings in the mask.

**Figure 10:**
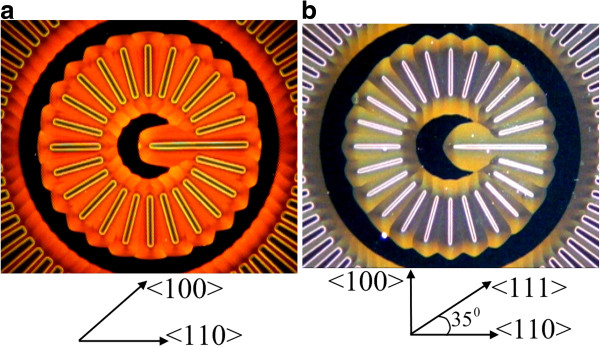
**Patterns formed in anodization through a ‘clock’ mask.** (**а**) On a (100) wafer at *I* = 400 mA and (**b**) on a (110) wafer at *I* = 20 mA.

Narrow strips circularly arranged in every 15° can also be a ‘source’ of por-Si propagating under the mask. Such a mask resembles a clock dial. Figure 10 shows patterns formed as a result of meeting of pore fronts from neighboring ‘hands.’ Their shape resembles a flower whose petals have more or less acute tips depending on the etching rate in the direction in which the hand points. Figure 10a for a (100) wafer clearly shows obtuse petals along four equivalent <110 > directions and acute petals along four <100 > directions turned by 45° with respect to <110 >. Similarly, for the Si (110) wafer (Figure 10b), the most acute petals are observed along the <100 > axis; the most obtuse, along <111>; and those of medium acuteness, along <110 > .

It should be noted that, despite the simplicity and clarity, patterns of this kind are rather inconvenient for obtaining quantitative characteristics, especially in the case of a weak anisotropy. Similarly to the beveled edge in measurements of small depths, the wagon-wheel procedure makes it possible, by extending the pattern, to raise the measured value by a factor of several tens and to record small differences in *w*.

The observed macroscopic anisotropy results from certain morphology of porous silicon, which is, in turn, produced by anisotropic formation of separate pores in silicon on the microscopic level, when the reaction predominantly occurs at the pore tip. The situation can be roughly described as follows: individual pores possess preferred growth directions and form main channels with dead-end branches. Depending on the angle between the crystallographic axis and the preferred motion direction of the main channels, the ‘road’ in the general front propagation direction (along the electric field) is more or less ‘wandering.’ It is the shape of the trajectory of separate pores and their diameter that, in the end, determine the propagation rate of the pore front.

According to the existing concepts [18], the anisotropy must be manifested most clearly when the pore formation rate is limited to the greatest extent by the kinetics of the chemical reaction, i.e., at low currents. The dissolution rate of silicon depends on the strength of its bonds, which varies between different crystallographic planes [17,18]. Direct silicon dissolution is known to demonstrate the most pronounced anisotropy [19]. Our data confirm that, at all the currents, the highest etching rate *V* of p^+^-Si is characteristic of the <100 > directions, whereas the <111 > direction is the slowest only at low currents: *V*111 < *V*112 < *V*110 < *V*113 < *V*100. The rates for the <112 > and <110 > axes swap places when the current was raised, and further increase in the current changes the slowest direction from <111 > to <110>. The general weakening of the anisotropy with increasing current density can be attributed to the increased role of oxidation and to growing importance of mass-transfer process inside pores.

## Conclusions

The wagon-wheel method is an effective tool for studying the etching rate anisotropy for mesoporous silicon, which can determine etching rates simultaneously for numerous crystallographic directions. This method can be well performed with the role of a mask played by a thin layer of n-type silicon on a p-Si substrate. This mask can be easily fabricated by local diffusion or ion implantation of a donor impurity. A study of how the anodization rate depends on current at various orientations demonstrated that, on the whole, the macroscopic anisotropy with respect to the dominating rate along the <100 > direction becomes weaker with increasing current density, and the relative rates for different directions change places. At high currents, the <110 > direction becomes the slowest, instead of <111>, and the rate along <112 > starts to exceed that along <110 > already at not-too-high currents.

## Abbreviations

por-Si: porous silicon; SEM: scanning electron microscopy.

## Competing interests

The authors declare that they have no competing interests.

## Authors’ contributions

EVA proposed the idea to apply the wagon-wheel technique for studying the anisotropy of porous silicon formation rate. She designed the photo-mask and developed the data processing methods. The paper was written by EVA. YAZ carried out the experimental part of the work, including photolithography, diffusion, and anodization of the samples. She performed most of the measurements, plotted the graphs, prepared pictures, and formatted the paper. All authors read and approved the final manuscript.

## References

[B1] Sato N Sakaguchi K Yamagata K Fujiyama Y Yonehara T Epitaxial growth on porous Si for a new bond and etchback silicon on insulator J Electrochem Soc 1995 142 3116 10.1149/1.2048698

[B2] Iyer SS Auberton-Herve AJ Silicon Wafer Bonding Technology for VLSI and MEMS Applications (EMIS Processing) 2002 London: Institution of Engineering and Technology 72 81

[B3] Brendel R Thin-film crystalline silicon mini-modules using porous Si for layer transfer Solar Energy 2004 77 969 10.1016/j.solener.2004.08.011

[B4] Gordon I Dross F Depauw V Masolin A Qiu Y Vaes J Van Gestel D Poortmans J Three novel ways of making thin-film crystalline-silicon layers on glass for solar cell applications Solar Energy Materials & Solar Cells 2011 95 S2

[B5] EU 7th Framework Programme Project R2M-Si. Roll to Module Siliconhttp://www.r2m-si.de

[B6] Künzner N Kovalev D Diener J Gross E Timoshenko VY Polisski G Koch F Fujii M Giant birefringence in anisotropically nanostructured silicon Optics Letters 2001 26 16 1265 10.1364/OL.26.001265 18049581

[B7] Künzner N Diener J Gross E Kovalev D Timoshenko VY Fujii M Form birefringence of anisotropically nanostructured silicon Phys Rev B 2005 71 195304 10.1364/ol.26.001265 18049581

[B8] Metzger TH Binder M Peisl J Canham L Properties of Porous Silicon Structure and morphology of porous silicon 1997 London: Institution of Engineering and Technology 112 117

[B9] Guendouz M Joubert P Sarret M Effect of crystallographic directions on porous silicon formation on patterned substrates Mat Sci Eng B 2000 69-70 43

[B10] Lehmann V Electrochemistry of Silicon 2002 Berlin: Wiley-VCH p 62 pp104-106

[B11] Uehara S Sugimoto J Yono D Matsubara T Micro-tip array fabrication by selective anodization of p + −type Si substrate Phys Stat Sol A 2003 197 275 10.1002/pssa.200306480

[B12] Uehara S Kubota K Nagaoka K Yoshida S Matsubara T Dependence of selective anodization characteristics on silicon substrate orientation Phys Stat Sol C 2005 2 3389 10.1002/pssc.200461180

[B13] Astrova EV Ulin VP Zharova YA Shul’pina IL Nashchekin AV Anisotropy effects in electrochemical etching of p+-Si J Electrochem Soc 2012 159 D172 10.1149/2.095203jes

[B14] Seidel H Csepregi L Heuberger A Baumgortel H Anisotropic etching of crystalline silicon in alkaline solutions I. Orientation dependence and behavior of passivation layers. J Electrochem Soc 1990 137 3612

[B15] Elwenspoek M Jansen HV Silicon Micromachining 2004 Cambridge: Cambridge University Press 16 18

[B16] Frey S Kemell M Carstensen J Langa S Foll H Fast pore etching Phys Stat Sol A 2005 202 1369 10.1002/pssa.200461104

[B17] Smith RL Collins SD Porous silicon formation mechanisms J Appl Phys 1992 71 R1 10.1063/1.350839

[B18] Kelly JJ Philipsen HGG Anisotropy in the wet-etching of semiconductors Current Opinion in Solid State and Materials Science 2005 9 84 10.1016/j.cossms.2006.04.003

[B19] Christophersen M Carstensen J Foll H Crystal orientation dependence of macropore formation in p-type silicon using organic electrolytes Phys Stat Sol A 2000 182 103 10.1002/1521-396X(200011)182:1<103::AID-PSSA103>3.0.CO;2-N

